# The complete mitochondrial genome of the Argentine red shrimp *Pleoticus muelleri* (Bate, 1888) (Crustacea, Decapoda, Solenoceridae)

**DOI:** 10.1080/23802359.2018.1511845

**Published:** 2018-10-26

**Authors:** Nack-Keun Kim, Jobaidul Alam, Gyung Ryul Kim, Sapto Andriyono, Hyun Park, Hyun-Woo Kim

**Affiliations:** a Interdisciplinary Program of Biomedical, Mechanical and Electrical Engineering, Pukyong National University, Busan, Republic of Korea;; b Fisheries and Marine Faculty, Universitas Airlangga, Surabaya, Indonesia;; c Korea Polar Research Institute, Korea Ocean Research and Development Institute, Incheon, Republic of Korea;; d Department of Marine Biology, Pukyong National University, Busan, Republic of Korea;

**Keywords:** Next generation sequencing, *Pleoticus muelleri*, mitochondrial genome, decapod, phylogeny

## Abstract

The complete mitochondrial genome of Argentine red shrimp, *Pleoticus muelleri* (Bate, 1888) was determined using the MiSeq platform. Its mitogenome (16,189 bp) encoded the canonical 13 protein-coding genes, 22 tRNA genes, two rRNA genes. Start codons of all protein-coding genes were ATN except for ATP8, while incomplete stop codons (T–) were identified in six genes including COXI, COXII, COXIII, NAD3, NAD5, and NAD6. The mitogenome of *P. muelleri* showed highest sequence identity with *Parapenaeopsis hardwickii* (82%) and *Solenocera crassicornis* (81%). Phylogenetic analysis showed three shrimps in Solenoceridae including *P. muelleri* were grouped together from those in Penaeidae, which suggests taxonomic reexamination for those in those family.

The Argentine red shrimp, *Pleoticus muelleri* is one of the most commercially imported shrimps in Argentina (Bertuche et al. 2005). This shrimp was distributed mainly in the Southwestern Atlantic Ocean from the coast of Santa Cruz, Argentina north to Rio de Janeiro, Brazil (Stamatopoulos 1993). Its bottom trawl fishery is now recommended to be prohibited to reduce the devastating impacts on benthic ecosystem with high bycatch as well as overfishing. Although this species is now exported in many countries, genetic study of *P. muelleri* has not been conducted (Batista et al. 2011; Azevedo and Barbosa 2016). As the first step, the full mitochondrial genome of *P. muelleri* was determined for the first time among the genus *Pleoticus*.

The frozen *P. muelleri* was purchased from an Argentina importer in July 2016 as the part of project to identify the origin of fisheries products in Korea. Its identification was confirmed by both morphological analysis and its 100% match of COI sequence to database (GenBank number MF490134). Examined specimen was stored at National Institute of Fisheries Science (NIFS). The full mitochondrial genome sequence was obtained by assembling two large PCR fragments (COI ∼ ND1: 10,386 bp, ND1 ∼ COI: 7377 bp) amplified by two primer sets designed from the multiple alignments of 13 decapod mitogenome sequences. The PCR products were pooled together in equal concentration and fragmented into 350 bp in length by Covaris^®^ M220 (Covaris Inc., Woburn, MA). A library was constructed by TruSeq^®^ RNA library preparation kit V2 (Illumina, San Diego, CA) and MiSeq Sequencer (Illumina, San Diego, CA) was used for NGS sequencing. Raw reads were trimmed and reads with low quality (QV < 20) were truncated at CLC Genomic Workbench v8.0 (CLC Bio, Cambridge, MA). Mitogenome was reconstructed with Geneious^®^ 11.0.2 (Kearse et al. 2012). The mitogenome of *Parapenaeopsis hardwickii* (KU302814) was employed as a reference sequence (Mao et al. 2016). The phylogenetic tree was constructed by MEGA 7 software (Kumar et al. 2016) with Minimum Evolution (ME) algorithm.

The mitochondrial genome of *P. muelleri* was 16,189 bp in length (GenBank Number: MH500232), which encodes 13 proteins, 22 tRNAs, and two ribosomal RNAs (12S and 16S ribosomal RNA) and a putative control region (D-loop). The proportions of A + T (68.22%) was much higher than G + C (31.78%). Total 10 genes were located at L strand and remaining 27 genes were at H strand. Overlapping protein-coding genes were detected between ATP8 ∼ ATP6 (6 bp) and ND4 ∼ ND4L (6 bp). Start codons of all protein-coding genes were ATN except for ATP8, while incomplete stop codons (T–) were identified in six genes including COXI, COXII, COXIII, NAD3, NAD5, and NAD6. Twenty-two tRNAs were predicted to be a typical folded clover-leaf secondary structure (Laslett and Canbäck 2008). As the result of phylogenetic analysis, *P. muelleri* was clustered together with *Parapenaeopsis hardwickii* (82%) and *Solenocera crassicornis* (81%) from those in family Penaeidae, which suggests further taxonomic reexamination of shrimps in superfamily Penaeoidea (Figure 1).

**Figure 1.**
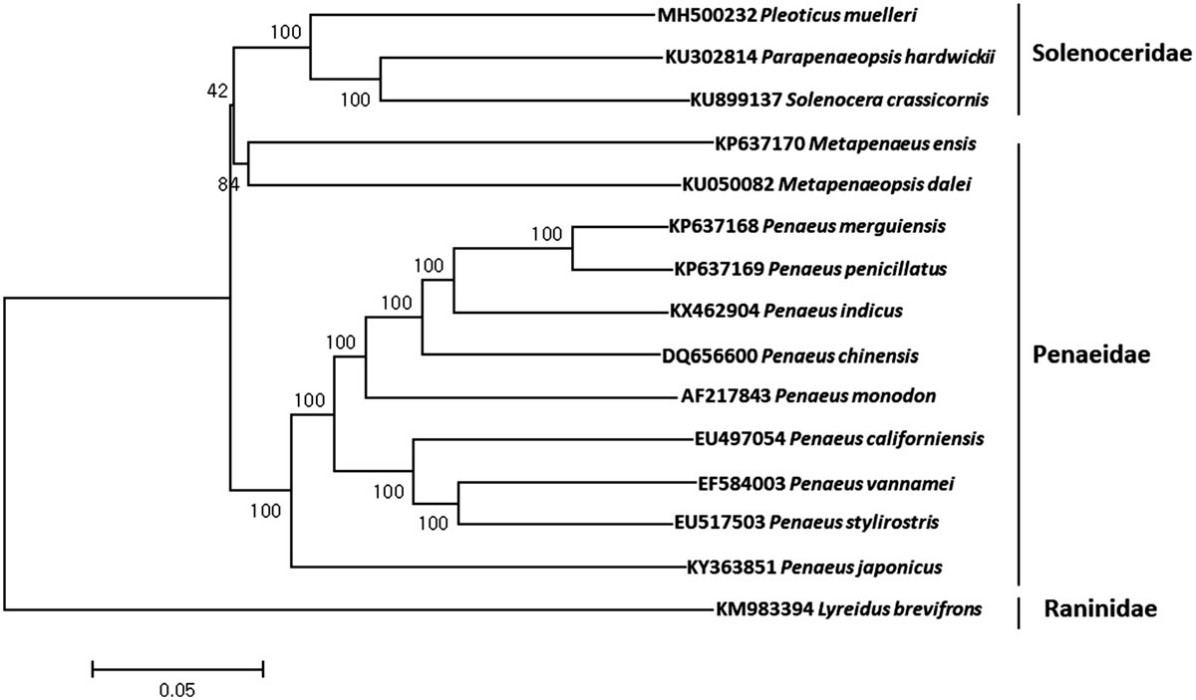
Phylogenetic tree of *Pleoticus muelleri* with other marine shrimps. The phylogenetic tree was constructed with the complete mitochondrial genome sequence of *Pleoticus muelleri* by using MEGA 7 software with Minimum Evolution (ME) algorithm with 1000 bootstrap replications. The mitochondrial genome sequences were obtained from the GenBank database.
